# Long-term outcomes of occipital nerve stimulation for chronic migraine: a cohort of 53 patients

**DOI:** 10.1186/s10194-016-0659-0

**Published:** 2016-07-30

**Authors:** Sarah Miller, Laurence Watkins, Manjit Matharu

**Affiliations:** 1 Headache Group, Institute of Neurology and The National Hospital for Neurology and Neurosurgery, Queen Square, London, WC1N 3BG UK; 2 Department of Neurosurgery, Institute of Neurology and The National Hospital for Neurology and Neurosurgery, Queen Square, London, UK

**Keywords:** Chronic migraine, Headache, Neurostimulation, Occipital nerve stimulation

## Abstract

**Background:**

Chronic migraine affects up to 2 % of the general population and has a substantial impact on sufferers. Occipital nerve stimulation has been investigated as a potentially effective treatment for refractory chronic migraine. Results from randomised controlled trials and open label studies have been inconclusive with little long-term data available.

**Methods:**

The long-term efficacy, functional outcome and safety of occipital nerve stimulation was evaluated in an uncontrolled, open-label, prospective study of 53 intractable chronic migraine patients.

**Results:**

Fifty-three patients were implanted in a single centre between 2007 and 2013. Patients had a mean age of 47.75 years (range 26–70), had suffered chronic migraine for around 12 years and had failed a mean of 9 (range 4–19) preventative treatments prior to implant. Eighteen patients had other chronic headache phenotypes in addition to chronic migraine. After a median follow-up of 42.00 months (range 6–97) monthly moderate-to-severe headache days (i.e. days on which pain was more than 4 on the verbal rating score and lasted at least 4 h) reduced by 8.51 days (*p* < 0.001) in the whole cohort, 5.80 days (*p* < 0.01) in those with chronic migraine alone and 12.16 days (*p* < 0.001) in those with multiple phenotypes including chronic migraine. Response rate of the whole group (defined as a >30 % reduction in monthly moderate-to-severe headache days) was observed in 45.3 % of the whole cohort, 34.3 % of those with chronic migraine alone and 66.7 % in those with multiple headache types. Mean subjective patient estimate of improvement was 31.7 %. Significant reductions were also seen in outcome measures such as pain intensity (1.34 points, *p* < 0.001), all monthly headache days (5.66 days, *p* < 0.001) and pain duration (4.54 h, *p* < 0.001). Responders showed substantial reductions in headache-related disability, affect scores and quality of life measures. Adverse event rates were favourable with no episodes of lead migration and only one minor infection reported.

**Conclusions:**

Occipital nerve stimulation may be a safe and efficacious treatment for highly intractable chronic migraine patients even after relatively prolonged follow up of a median of over 3 years.

**Electronic supplementary material:**

The online version of this article (doi:10.1186/s10194-016-0659-0) contains supplementary material, which is available to authorized users.

## Background

Chronic migraine (CM) is a highly disabling primary headache disorder affecting approximately 2 % of the general population [13, 27]. Chronic migraine is diagnosed when a patient has headaches on at least 15 days per month, of which at least 8 days meet diagnostic criteria for migraine [13]. Compared to episodic migraine, CM sufferers report higher levels of headache related disability and comorbid psychiatric disorders, reduced rates of productivity at work or school and impaired health-related quality of life [2, 3, 15, 23]. The annual direct cost of chronic migraine is estimated to be around €1800 in Europe and between $3500–$4150 in the United States [7, 25]. Besides the costs of medical treatment the associated indirect and socioeconomic costs are huge with one recent web-based study reporting total annual costs of over $8000 amongst CM patients [4, 24].

Treatment for CM is based on prophylactic medication aimed at reducing the frequency and severity of migraine headaches. Despite best medical practice, it is estimated that around 5 % of CM patients seen in headache clinics will prove refractory to treatment [31]. The definition of refractory chronic migraine is not yet concrete, although European Headache Federation guidelines propose that patients should meet diagnostic criteria for CM, have failed adequate trials of at least three preventive drugs, alone or in combination (from beta-blockers, anticonvulsants, tricyclic antidepressants, flunarizine or candesartan, and OnabotulinumtoxinA), and have received multidisciplinary input for any psychiatric co-morbidities [21]. For this group of patients, occipital nerve stimulation (ONS) has been carried out with some promising results in both open-label and controlled-trial evidence. However, results of the placebo-controlled trials (all with follow-up periods of just 3-months) have been interpreted with caution due to contradictory outcomes and high adverse event rates. The ONSTIM (occipital nerve stimulation for the treatment of intractable chronic migraine headache) feasibility trial quoted a responder rate of 39 % in the active vs. 6 % in the sham group, yet the PRISM (precision implantable stimulator for migraine) study conducted soon after failed to show any significant difference between active and sham stimulation [17, 30]. The largest sham-controlled study of ONS in CM, consisting of 157 patients undergoing ONS for 3 months also failed to show a significant difference in its primary end-point of a 50 % reduction in pain intensity between sham and active treatment. However, when this study examined a 30 % reduction in end-point, a level representing a “much improved” state in chronic pain research, a significant difference between groups was observed [11, 34]. Open-label data has been more positive with response rates of around 56 % although follow-up periods have been relatively short [18].

With sparse data on follow-up past 12 months of ONS implant, there is a need for studies examining the sustained effects of treatment. This uncontrolled, open-label prospective observational study reports on the long-term outcomes of a single-center cohort of 53 patients with CM undergoing ONS.

## Methods

### Patients

Patients with medically intractable chronic migraine seen in the headache clinic at the National Hospital for Neurology and Neurosurgery, Queen Square, London, UK were offered ONS. Patients were reviewed and operated on by a single multidisciplinary headache team and were implanted over a 6-year period from March 2007 to December 2013. All patients fulfilled the International Classification of Headache Disorders (ICHD) 2nd edition and revised ICHD-3beta diagnostic criteria for CM and also proposed criteria for intractable chronic migraine [12, 14, 21], although, given the time period of the study not all patients had received OnabotulinumtoxinA as recommended in the recent European Headache Federation guidelines due to the fact that it was not approved for use in the UK National Health Service until 2012 [21, 26]. Under the supervision of our institution’s Clinical Effectiveness Supervisory Committee (CESG) with arrangements for clinical governance, consent and audit, we offered ONS to patients with medically intractable CM. The procedure was provided on the basis of a “humanitarian intervention”. In addition, ethics board approval for data collection and publication was granted by Northwick Park Hospital Research Ethics Committee, Hampstead, London, UK.

### Surgical procedure

Bilateral ONS electrodes, leads and an implantable pulse generator (IPG) were implanted in all patients (Table 1). Systems from both Medtronic (*n* = 47) and St Jude Medical (*n* = 6) were utilized with octad electrodes used in 51 patients and quad electrodes in two. The patient was placed into the lateral position and a midline posterior cervical incision made. Initially, the insertion point of the electrodes was the spinous process of C1, passing superior and laterally, using a curved Tuohy needle and an image intensifier to aid positioning. This method evolved over time so that implantation level was aimed at the greater occipital nerve as it emerged superior to the nuchal line. In this amended technique, the electrode was passed using a blunt plastic tube to limit the risks of the electrode tip being tunneled too close to the skin. The evolution of surgical technique occurred in response to adverse events such as recruitment of neck muscles during stimulation or erosion of the electrode tip through the scalp. Given that both techniques target the same nerve it is felt unlikely that the implant technique would directly account for changes in efficacy. Electrodes were looped and anchored to cervical fascia and then tunneled to a lateral cervical or infraclavicular skin crease intermediate incision. An infraclavicular or abdominal incision was made (according to patient preference) and a pocket formed into which the IPG was placed. Electrodes were tunneled to the intermediate incision site where a pair of extension leads were connected. Silicone sheaths were used to protect lead connections. Topical gentamicin was introduced around the pocket prior to closure. Our unit did not employ trial stimulation as it was felt that the current evidence to support its use is outweighed by the risks of extra surgical procedures.

**Table 1:** Information on the occipital nerve stimulator systems implanted

	*N* = 53
ONS Manufacturer	
Medtronic	47 (88.6 %)
St Jude	6 (11.3 %)
IPG	
Standard	6 (11.3 %)
Rechargeable	24 (45.3 %)
Standard changed to rechargeable	23 (43.4 %)
Electrodes	
Quad	2 (3.8 %)
Octad	51 (96.2 %)
IPG Site	
Abdomen	21 (39.36 %)
Infraclavicular	23 (43.4 %)
Abdomen moved to infraclavicular	9 (17.0 %)

*IPG* implantable pulse generator, *ONS* occipital nerve stimulator

At initial programming, frequency was set at 60Hz with a pulse width of 240 μs. Polarity of the electrodes was adjusted during follow up visits to ensure comfortable bilateral paresthesia in the bilateral occipital region. Patients were provided with remote controls allowing them to adjust their stimulation amplitude but were asked to use continuous stimulation where possible. Stimulation settings and changes were recorded at each visit. Medications were changed as needed at the discretion of the headache specialist.

### Data collection

Data were collected prospectively and entered onto a clinical database (Microsoft Excel, Microsoft Corporation, Redmond, WA, USA). Data including demographics, diagnosis, daily pain severity and duration, previous and current treatments, and adverse events were recorded. Patients were reviewed in clinic every 3 months for the first year and then every 6 to 12 months thereafter. Patients prospectively completed headache diaries recording pain severity on a verbal rating scale (VRS; 0 = no pain to 10 = extreme pain) and daily pain duration (in hours) for 1 month prior to implant and 2 weeks prior to each follow-up visit. This 2-week data was used to calculate the mean monthly moderate-to-severe headache days (days on which pain of VRS ≥ 4 lasting at least 4 h), mean monthly headache days (days on which any pain was recorded), mean daily pain severity and mean daily hours of pain over these periods of time. Where multiple headache types were present, patients were asked to differentiate between these and separate diaries completed for each to allow the outcome of each phenotype to be established (examples of the headache diaries used to record chronic migraine are included in the Additional file 1: Figure S1).

Migraine Disability Assessment Scores (MIDAS) and Headache Impact Test 6 Scores (HIT-6), both validated for their use in migraine, were recorded pre- and post-ONS to monitor headache related disability. Euro-QoL (Euro-QoL 5D index [EQ-5D] and Euro-QoL visual analogue score [EQ-VAS]), Short Form 36 Questionnaires (SF36), Beck Depression Inventory II (BDI-II), Hospital Anxiety (HAD-A) and Hospital Depression (HAD-D) Scores were used to monitor quality of life and mental state pre- and post-implant. Patients were asked to provide a subjective global estimate of improvement in their migraine headaches from 0 to 100 % at follow-up.

Details of any adverse events were recorded throughout follow-up as they occurred. Events were categorized as “hardware related” if they involved problems with the device components, “biological” if they were reactions to the device or surgical procedure and “stimulation related” if they involved stimulation issues [34].

The primary outcome measure was the improvement in mean monthly moderate-to-severe headache days at final follow up compared to the baseline. A responder was defined as a patient who had a 30 % or more reduction in mean monthly moderate-to-severe headache days. Secondary outcome measures included changes in monthly moderate-to-severe headache days at each time point, mean monthly headache days, mean daily pain intensity, mean daily pain duration, headache-related disability scores, affective measures and quality of life scores. Adverse events were also examined.

### Statistics

All statistical analyses were conducted using IBM SPSS Statistics version 22 (IBM Corp. Int.). A last observation carried forward technique was used in the case of missing data. Descriptive statistics were summarized as appropriate. Data is presented as mean ± standard deviation (SD), range and frequencies. Paired and independent *t*-tests were used to compare treatment effect as appropriate. All statistical tests were two-sided with a significance level of 95 % and are presented with 95 % confidence intervals.

## Results

### Patient demographics

Fifty-three patients (37 female) with intractable CM underwent bilateral ONS insertion between March 2007 and December 2013 (Table 2). The mean age at implant was 47.75 years (±11.48). Patients had suffered chronic migraine for a mean of 11.77 years (±10.90). The cohort had failed a mean of 9.36 (±2.61) preventative medications prior to implant (Table 3). Only 22.6 % of patients had reported a previous response to greater occipital nerve block (response defined as a more than 50 % reduction in headache severity or frequency lasting at least 2 weeks).

**Table 2:** Demographic data

Age	
Mean (SD)	47.75 years (±11.48)
Range	26–70 years
Sex	
Male	16 (30.2 %)
Female	37 (69.8 %)
Laterality	
Unilateral	33 (62.3 %)
Bilateral	20 (37.7 %)
Aura	28 (52.9 %)
Visual	22 (41.5 %)
Sensory	15 (28.3 %)
Hemiplegic	6 (11.3 %)
Speech	5 (9.4 %)
Duration from onset of migraine	
Mean (SD)	31.51 years (±14.52)
Range	5–58 years
Duration from onset of Chronic Migraine	
Mean (SD)	11.77 years (±10.90)
Range	3–48 years
Co-existent headache types	18 (33.9 %)
Chronic cluster headache	10 (18.7 %)
SUNCT/SUNA	5 (9.4 %)
Chronic cluster headache + SUNCT/SUNA	2 (3.8 %)
SUNCT/SUNA + hemicrania continua	1 (1.9 %)
Number of headache types	
1	35 (66.0 %)
2	15 (28.3 %)
3	3 (5.7 %)
Monthly days of acute medication	
Mean (SD)	11.77 (±10.34)
Range	0–30
Medication overuse at implant	20 (37.7 %)
Mean number of preventatives prior to ONS	
Mean (SD)	9.36 (±2.61)
Range	4–19
Prior response to GON block	12 (22.6 %)
Follow up since ONS implant	
Median	42.00 months
Mean (SD)	46.79 months (±21.70)
Range	6–97 months

*GON* greater occipital nerve, *ONS* occipital nerve stimulation, *SD* standard deviation, *SUNA* short lasting unilateral neuralgiform headache attacks with autonomic features, *SUNCT* short lasting unilateral neuralgiform headache attacks with conjunctival injection and tearing

**Table 3:** Medications taken for chronic migraine prior to occipital nerve stimulation

	Number of patients who tried drug (% of cohort *n* = 53)	Daily dose range (mg)	Mean maximum daily dose (mg)
Beta-Blockers	42 (79.2 %)	30–320	140.80
Topiramate	49 (92.4 %)	25–400	170.65
Sodium Valproate	47 (88.6 %)	300–2500	1047.50
Gabapentin	51 (96.2 %)	300–3600	2206.97
Pregabalin	32 (60.3 %)	50–600	384.16
Flunarizine	36 (67.9 %)	5–20	8.79
Pizotifen	45 (84.9 %)	1.5–4.5	2.39
Methysergide	44 (83.0 %)	1–12	6.10
Tricyclic Antidepressant:	51 (96.2 %)	–	–
Amitriptyline	44 (83.0 %)	10–150	56.31
Dosulepin	28 (52.8 %)	25–225	101.73
NSAID	42 (79.2 %)	–	–
Acupuncture	3 (5.7 %)	–	–
Botox	7 (13.2 %)	–	–
IV DHE	45 (84.9 %)	–	–
GONB	53 (100 %)	–	–

*Botox* OnabotulinumtoxinA, *DHE* Dihydroergotamine, *GONB* greater occipital nerve block, *IV* intravenous, *NSAID* non-steroidal anti-inflammatory drug

Eighteen patients (35.3 %) reported other headache phenotypes in addition to chronic migraine: ten with chronic cluster headache; five with short lasting unilateral neuralgiform headache attacks; two with chronic cluster headache and short lasting unilateral neuralgiform headache attacks; and, one with short lasting unilateral neuralgiform headache attacks and hemicrania continua (Table 2). All kept separate diaries for each phenotype throughout the follow-up period (Additional file 1: Figure S1).

Twenty patients (37.7 %) were overusing acute medications at time of implant (as defined by ICHD-3b criteria). All of those with CM alone had previously undergone a medication withdrawal as part of their routine clinical treatment and failed to report any significant improvement in their headaches, thereby excluding medication overuse headache.

### Whole cohort

**Fig. 1:**
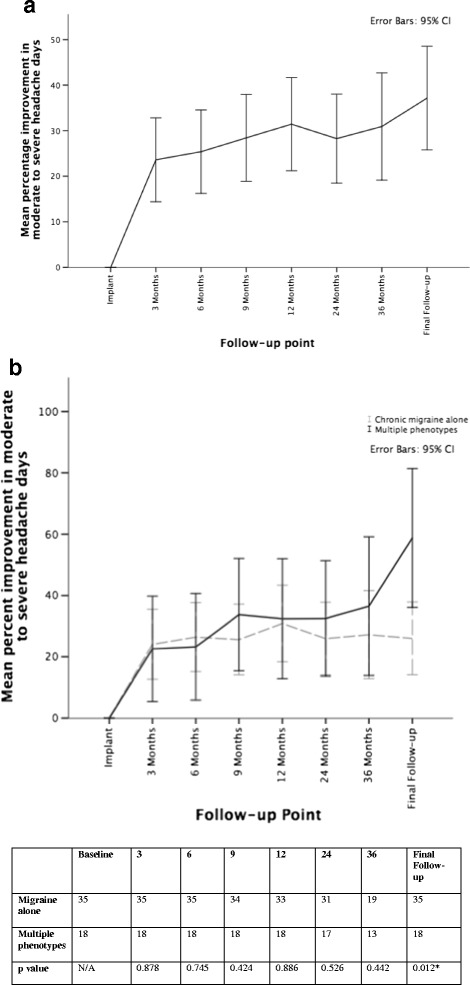
Changes in moderate-to-severe headache days following occipital nerve stimulation. **a** Improvement of moderate-to-severe headache days of whole cohort over follow-up period. **b** Improvement in moderate-to-severe headache days of those with chronic migraine alone compared to those with multiple phenotypes over follow-up period. Table provides number of subjects included at each time point

Median follow-up time was 42.00 months with a range of 6–97 months. At follow-up, five patients had had their ONS devices removed due to lack of efficacy and a further three had the device switched off for the same reasons. There was no significant difference in the follow-up time of responders and non-responders (*p* = 0.619). The primary outcome of a 30 % or more reduction in moderate-to-severe headache days was observed in 45.3 % (*n* = 24) at final follow-up. Monthly moderate-to-severe headache days fell by 8.51 days (95%CI 5.63, 11.38; *p* < 0.001) a reduction of 37.1 %. Figure 1a shows the change in moderate-to-severe headache days over the follow-up period. A reduction of 50 % or more in monthly moderate-to-severe headache days was seen in 37.7 % (*n* = 20). Significant reductions were seen in mean any-headache days (−5.66), mean daily pain duration (−4.54 h) and mean daily pain intensity (−1.34 points on VRS) (Table 4). Although a significant reduction of 3.94 points was recorded in HIT-6, the reduction in MIDAS was not significant (−20.62). Affect scores, EQ5D and SF-36 composite scores failed to show any improvement across the cohort but the Euro-VAS did show significant improvement (Table 5).

**Table 4:** Summary of efficacy outcome measures of occipital nerve stimulation for chronic migraine

Outcome measure	Prior to ONS	Post-ONS	Percentage change	Mean change (95 % CI)	*p* Value
Whole cohort (*n* = 53)					
Headache days^a^ (±SD)	29.57 (±2.12)	23.91 (±10.04)	20.0 % (±32.91)	5.66	< 0.001*
Range	18–30	0–30	0–100	(3.07, 8.25)	
Moderate-to-severe headache days^a^ (±SD)	26.51 (±6.48)	18.00 (±12.79)	37.1 % (±41.27)	8.51	< 0.001*
Range	5–30	0–30	0–100	(5.63, 11.38)	
Average daily pain intensity VRS (±SD)	6.00 (±1.71)	4.66 (±2.59)	27.8 % (±32.87)	1.34	< 0.001*
Range (VRS)	3–9	0–10	0–100.0	(0.64, 2.03)	
Average daily headache hours (±SD)	16.06 (±5.38)	11.52 (±7.12)	31.6 % (±37.12)	4.54	< 0.001*
Range (hours)	2–24	0–24	0–100	(2.62, 6.45)	
Mean Patient estimated benefit (±SD)		31.7 % (±33.12)			
Range		0–100			
Chronic migraine alone (*n* = 35)					
Headache days^a^ (±SD)	29.34 (±18–30)	26.23 (±8.26)	12.0 % (±26.29)	3.11	< 0.001*
Range	18–30	2–30	0–93	(0.79, 5.43)	
Moderate-to-severe headache days^a^ (±SD)	26.83 (±6.74)	21.03 (±11.33)	26.0 %(±34.58)	5.80	0.010*
Range	5–30	0–30	0–100	(2.76, 8.83)	
Average daily pain intensity VRS (±SD)	6.09 (±1.63)	4.89 (±2.34)	23.7 % (±28.40)	1.20	0.003*
Range (VRS)	3–9	0–9	0–100	(0.43, 1.96)	
Average daily headache hours (±SD)	15.10 (±4.15)	12.44 (±6.18)	24.5 % (±34.24)	2.75	0.003*
Range (hours)	2.0–24.0	0–24.0	0–100	(0.99, 4.50)	
Mean Patient estimated benefit (±SD)		30.0 % (±29.77)			
Range		0–95			
Multiple headache phenotypes (*n* = 18)					
Headache days^a^ (±SD)	30.00 (±0.0)	19.39 (±11.80)	35.3 % (±39.37)	10.61	0.001
Range	30	0–30	0–100	(4.73, 16.48)	
Moderate-to-severe headache days^a^ (±SD)	25.94 (±5.90)	13.78 (±13.97)	58.7 % (±45.48)	12.16	< 0.001
Range	10–30	0–30	0–100	(6.63, 17.69)	
Average daily pain intensity VRS (±SD)	5.83 (±1.88)	4.22 (±3.04)	23.7 % (±28.40)	1.61	0.039
Range (VRS)	3–9	0–10	0–100	(0.09, 3.12)	
Average daily headache hours (±SD)	17.72 (±6.96)	9.80 (±8.53)	45.4 % (±39.56)	7.91	0.001
Range (hours)	5.0–24.0	0.0–24.0	0–100	(3.62, 12.21)	
Mean Patient estimated benefit (±SD)		35.2 % (±39.53)			
Range		0–100			

*CI* confidence interval, *ONS* occipital nerve stimulation, *SD* standard deviation, *VRS* verbal rating scale

*P value less than 0.05; ^a^time period one month

**Table 5:** Headache related disability, affect and quality of life scores following occipital nerve stimulation

	Pre ONS (*n* = 53)	Post ONS (*n* = 53)	Mean Change in Score (95 % CI)	*p*-value
Whole cohort (*n* = 53)				
MIDAS				0.188
Mean (±SD)	154.91 (±84.03)	134.28 (±92.70)	20.62	
Range	18–270	0–270	(−10.41, 51.65)	
HIT-6				0.009*
Mean (±SD)	69.17 (±6.88)	65.23 (±9.27)	3.94	
Range	52–98	36–89	(1.04, 6.84)	
HAD-A				0.618
Mean (±SD)	10.34 (±4.46)	9.96 (±4.86)	0.377	
Range	2–21	0–19	(−1.13, 1.88)	
HAD-D				0.127
Mean (±SD)	11.36 (±4.23)	10.26 (±5.40)	1.09	
Range	1–20	0–20	(−0.32, 2.51)	
BDI-II				0.132
Mean (±SD)	26.11 (±11.07)	23.13 (±13.59)	2.98	
Range	2–46	0–59	(−0.93, 6.89)	
SF-36 Physical Composite				0.054
Mean (±SD)	27.12 (±8.16)	29.41 (±11.43)	−2.29	
Range	11.1–15.9	12.1–55.7	(−4.62–0.04)	
SF-36 Mental Composite				0.076
Mean (±SD)	34.72 (±11.54)	37.97 (±13.25)	−3.25	
Range	19.6–62.5	9.8–61.2	(−6.84–0.35)	
EQ5D				
Mean (±SD)	0.66 (±0.11)	0.64 (±0.15)	0.02	0.317
Range	0.26–0.83	0.25–1.0	(−0.16–0.05)	
EQ-VAS				
Mean (±SD)	40.51 (±21.09)	49.78 (±25.00)	9.27	0.009*
Range	0–90	5–95	(−16.09– −2.43)	
Chronic migraine alone (*n* = 35)				
MIDAS				
Mean (±SD)	162.17 (±86.50)	135.97 (±91.33)	26.20	0.194
Range	18–270	0–270	(−13.95,66.35)	
HIT-6				
Mean (±SD)	69.91 (±6.72)	66.26 (±8.14)	3.65	0.038*
Range	61–98	36–78	(0.21, 7.10)	
HAD-A				
Mean (±SD)	10.69 (±4.33)	10.83 (±4.42)	−0.14	0.864
Range	2–21	2–18	(−1.82, 7.10)	
HAD-D				
Mean (±SD)	11.91 (±4.09)	11.34 (±5.01)	0.57	0.396
Range	3–20	1–19	(−0.78, 1.92)	
BDI-II				
Mean (±SD)	27.09 (±11.22)	24.37 (±13.03)	2.71	0.199
Range	2–45	0–48	(−1.49, 6.92)	
SF-36 Physical Composite				
Mean (±SD)	27.71 (±8.11)	27.69 (±10.58)	0.02	0.986
Range	11.10–45.90	12.10–55.0	(−2.6, 2.73)	
SF-36 Mental Composite				
Mean (±SD)	32.71 (±11.20)	36.86 (±12.72)	−4.14	0.039*
Range	19.60–62.50	9.80–61.20	(−80.7, −0.22)	
EQ5D				
Mean (±SD)	0.56 (±0.12)	0.62 (±0.15)	0.03	0.105
Range	0.26–0.83	0.25–0.83	(−0.01, 0.07)	
EQ-VAS				
Mean (±SD)	42.43 (±22.44)	48.64 (±22.87)	−6.21	0.057
Range	10–90	10–90	(−12.63, 0.20)	
Multiple phenotypes (*n* = 18)				
MIDAS				
Mean (±SD)	140.78 (±79.46)	131.00 (±97.89)	9.77	0.701
Range	24–270	0 + 270	(−42.96, 62.52)	
HIT-6				
Mean (±SD)	67.72 (±7.16)	63.22 (±11.14)	4.50	0.122
Range	52–78	42–89	(−1.33, 10.33)	
HAD-A				
Mean (±SD)	9.67 (±4.75)	8.28 (±5.36)	1.38	0.377
Range	3–18	0–19	(−1.84, 4.62)	
HAD-D				
Mean (±SD)	10.44 (±4.69)	8.17 (±5.63)	2.27	0.178
Range	1–19	0–20	(−1.14, 5.69)	
BDI-II				
Mean (±SD)	24.22 (±10.85)	20.72 (±14.68)	3.50	0.415
Range	6–46	0–59	(−5.33, 12.33)	
SF-36 Physical Composite				
Mean (±SD)	26.85 (±7.59)	36.36 (±12.55)	−5.91	0.010*
Range	13.70–42.50	12.70–55.70	(−10.23, −1.58)	
SF-36 Mental Composite				
Mean (±SD)	36.36 (±11.24)	40.13 (±14.35)	−3.77	0.329
Range	20.10–59.40	15.30–59.50	(−11.68, 4.14)	
EQ5D				
Mean (±SD)	0.65 (±0.85)	0.67 (±0.13)	−0.14	0.619
Range	0.54–0.83	0.41–1.00	(−0.77, 0.04)	
EQ-VAS				
Mean (±SD)	37.35 (±18.88)	51.65 (±28.81)	−14.29	0.069
Range	10–70	5–95	(−29.85, 1.26)	

BDI-II, Becks Depression Inventory; CI, confidence interval; EQ-VAS, Euro-QoL visual analogue score; EQ5D, Euro-QoL 5D Index; HAD-A, Hospital Anxiety and Depression scores-anxiety specific; HAD-D, Hospital Anxiety and Depression scores – depression specific; HIT-6, Headache Impact Test; MIDAS, Migraine Disability Assessment Scale; ONS, occipital nerve stimulation; SD (Standard deviation); SF-36, Short Form 36

Clinical non-responders failed to show any improvement in any-headache days, severity or duration, headache related disability, quality of life or affect scores. Responders showed significant improvements in HIT-6 (−7.75, *p* = 0.009), HAD-D (−2.98, *p* = 0.012), BDI-II (−7.04 points, *p* = 0.012), Euro-VAS (42.93 points, *p* < 0.001) and both SF-36 Physical (4.59 points, *p* = 0.017) and Mental Composite scores (5.12 points, *p* = 0.034). In responders, significant improvements were also observed in headache days (−11.50, *p* < 0.001), pain severity (−2.75 points, *p* < 0.001) and daily pain hours (−8.60, *p* < 0.001).

Patient estimated improvement in their migraine at final follow-up was 31.7 % (±33.12) and 23 (46 %) would recommend the device to others. For responders, estimated improvement was 40.0 % (±38.27) and in non-responders 15.0 % (±24.54).

**Fig. 2:**
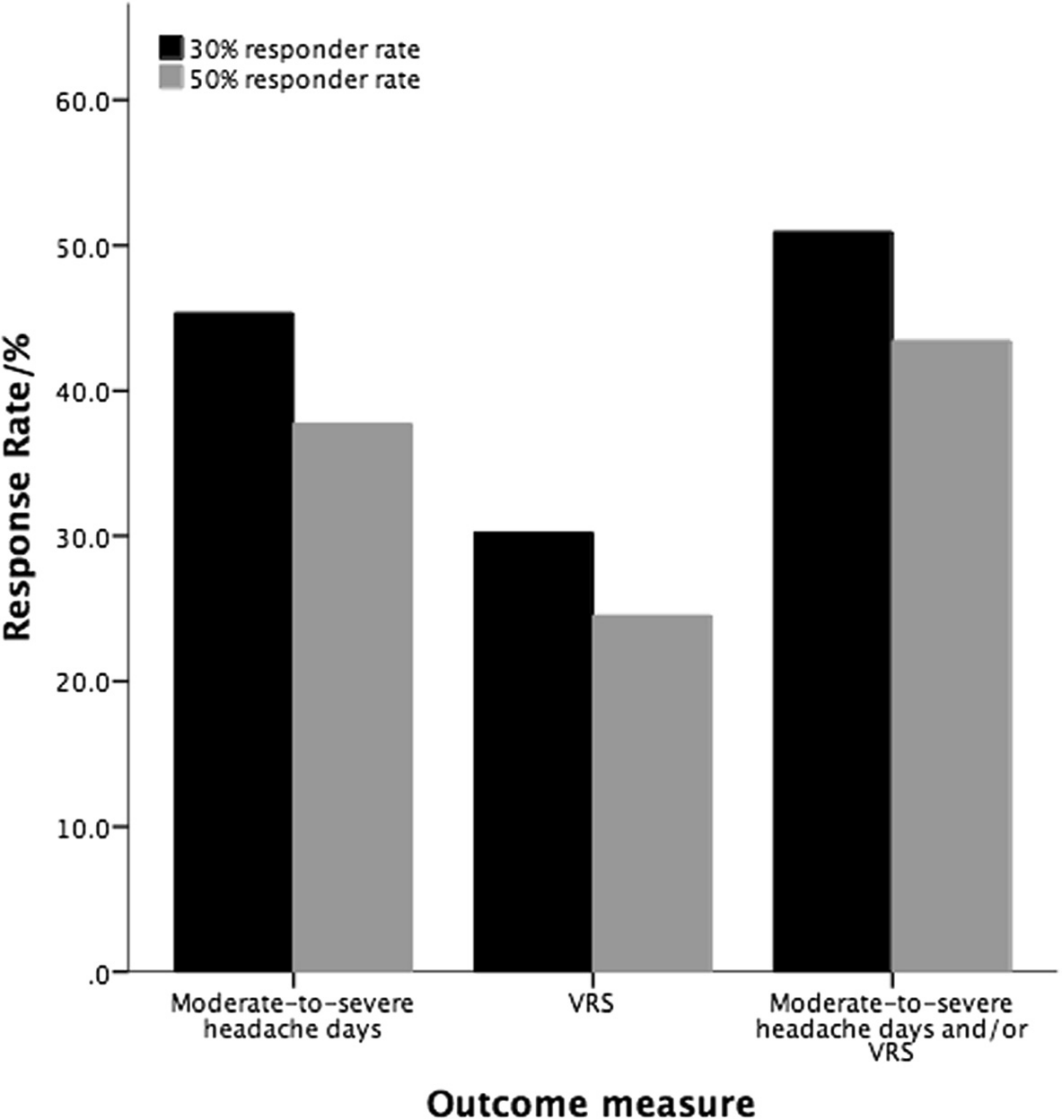
Responder rates of all chronic migraine patients to prolonged occipital nerve stimulation treatment by outcome measure. Various outcome measures have been used across the occipital nerve stimulation literature to measure response in chronic migraine. The response rate of the cohort is shown for each of these outcome measures – headache days, pain intensity and a combination of headache days and/or pain intensity. *VRS* verbal rating scale

Responder rates of changes in pain intensity and combinations in headache frequency and severity are summarized in Fig. 2.

### Chronic migraine alone

In the 35 patients with CM alone, the median follow-up time was 39.00 months. A 30 % or more reduction in moderate-to-severe headache days was observed in 36.4 % (*n* = 12) at final follow-up. Monthly moderate-to-severe headache days fell by 5.80 days (95%CI 2.76, 8.83; *p* = 0.010) a reduction of 26.0 %. Figure 1b shows the change in monthly moderate-to-severe headache days over time. The average time to reach a 30 % improvement (calculated using diary scores at each time point available) was 7.05 months (±6.47). A reduction of at least 50 % in moderate-to-severe headache days was seen in 27.3 % (*n* = 9). Significant reductions were seen in mean any-headache days (−3.11 days), mean daily pain duration (−2.75 h) and mean daily pain score (−1.20 points on VRS) (Table 4). Although a reduction was seen in MIDAS scores at final follow-up this was not significant (−26.20 points). The HIT-6 score did show a significant reduction, however (−3.65 points) (Table 5). Affect scores did not show significant changes. Quality of life scores showed significant improvement in SF36 Mental composite scores (4.14 points) but not in EQ-5D, EQ-VAS or SF-36 Physical composite scores (Table 5).

### Multiple phenotypes including CM

In those 18 patients with multiple headache types including CM, the median follow-up time was 45.00 months (range 17–87). The 30 % response rate was 66.7 % (*n* = 12) which is significantly greater than the response rate of those with CM alone at final follow-up (*p* = 0.012). However, at no other time-point was a significant difference seen between those with single vs. multiple headache types (Fig. 1b). Monthly moderate-to-severe headache days fell by 12.16 days (95 % CI 6.63, 17.69; *p* < 0.001) a reduction of 58.7 %. The average time to reach a 30 % improvement was 5.33 months (±2.74). A 50 % or more reduction in moderate-to-severe headache days was seen in 61.1 % (*n* = 11). Significant reductions were seen in mean any-headache days (−10.61 days), mean daily pain duration (−7.91 h) and daily pain intensity (−1.61 points on VRS) (Table 4). Neither MIDAS nor HIT-6 showed any significant change (Table 5). Affect scores, EQ5D, EQ-VAS and SF-35 Mental composite scores failed to show any improvement but SF-36 Physical composite showed significant improvement at final follow-up.

In CM responders, 6/9 CCH, 3/4 short lasting unilateral neuralgiform headache attacks and 1/1 hemicrania continua responded to ONS (defined as a more than 50 % reduction in daily attack frequency for CCH and short lasting unilateral neuralgiform headache attacks, and a more than 30 % reduction in moderate-to-severe daily headache days for hemicrania continua). In CM non-responders, 2/3 CCH and 4/5 short lasting unilateral neuralgiform headache attacks responded to ONS.

### Acute medication use

The mean number of days on which patients used any acute medication fell by 2.43 days (*p* = 0.154). There was no change in the proportion of patients overusing acute medication pre- and post-treatment (37.7 v. 35.8 %) (*p* = 0.840). The proportion recording acute medication overuse prior to implant did not differ in responders and non-responders (55 v 27 %; *p* = 0.078).

### Preventative medication use

Twenty-three patients were taking at least one preventative medication at implant. Following ONS, six patients (26.1 %) had stopped all medications, four (17.4 %) had reduced the dose of or stopped at least one medication, eight (34.8 %) had had no change in their medication doses and five (21.7 %) had increased the dose or number of medications taken.

### Time to effect and recurrence of attacks

The median time of the whole cohort to reach a 30 % improvement in moderate-to-severe headache days was 5.50 months (range 1–24 months). In responders, the median time was 4.00 months. Although a significant difference was seen in moderate-to-severe headache days between baseline and three months in responders (7.70 days, *p* < 0.001), no such change was seen between months 3 and 6 (*p* = 0.705), 3 and 9 (*p* = 0.498) or 3 and 12 months (*p* = 0.918).

Twenty-one subjects had their ONS turned off for a period of time – 13 due to battery depletion, six due to lack of efficacy and two due to ONS technical issues. In 15 patients, migraine pain worsened when the ONS was off. The mean time to pain worsening was 2.47 (range 1–6 months). There was no difference in 30 % response rate of those who had their ONS switched off temporarily at any point (*p* = 0.777).

### Stimulation settings

Mean stimulation amplitude was 1.46 V (range 0.29–3.95), pulse width 449.90 μs (range 370–570) and frequency 72.13Hz (range 50–140).

### Adverse events

Adverse events were categorized as “hardware related” if they involved problems with the device components, “biological” if there were reactions to the device or surgical procedure and “stimulation related” if they involved stimulation issues (Table 6). In total, 54 events were recorded in 26 patients. Twenty-two hardware issues were recorded including ten system revisions (18.9 %), five explantations secondary to lack of efficacy (9.4 %) and four battery depletions in under 1 year (7.5 %). Three electrode erosions (5.7 %) were seen, none associated with infection, which all required surgical intervention. No episodes of lead migration or fracture were recorded. One episode of infection of a wound site was observed that received medical management only.

**Table 6:** Summary of adverse events of prolonged follow-up of ONS for chronic migraine

	Adverse event	Surgical intervention	Medical management	Total events
Hardware related	Lead migration	0	0	0
	Lead fracture	0	0	0
	Electrode erosion	3 (5.7 %)	0	3 (5.7 %)
	ONS system revision	10 (18.9 %)	0	10 (18.9 %)
	Change to rechargeable system	9 (17.0 %)		
	Secondary to lead tethering	1 (1.9 %)		
	Explantation (Due to efficacy)	5 (9.4 %)	0	5 (9.4 %)
	Battery depletion: (Failure in under one year)	4 (7.5 %)	0	4 (7.5 %)
Total hardware related events				22
Biological	Infection	0	1 (1.9 %)	1 (1.9 %)
	Pain over IPG/lead/wound sites	1 (1.9 %)	5 (9.4 %)	6 (11.3 %)
	Neck stiffness	0	8 (15.1 %)	8 (15.1 %)
	Allergy to surgical material	0	1 (1.9 %)	1 (1.9 %)
	Wound site complication	0	2 (3.8 %)	2 (3.8 %)
Total biological related events				18
Stimulation associated	Undesirable changes in stimulation	0	14 (26.4 %)	14 (26.4 %)
Total stimulator associated events				14
Total		54 events (involving 26 patients)		

*IPG* implantable pulse generator, *IV* intravenous, *ONS* Occipital nerve stimulator

## Discussion

Weiner and Reed were the first to report the potential use of ONS for intractable occipital neuralgia [36]. Subsequent review and imaging of the patients by headache specialists, however, suggested many of these patients actually had CM [22]. There have now been three placebo-controlled studies on ONS in CM and although their results have been somewhat contradictory recent meta-analysis suggests an overall positive effect of treatment [17, 30, 34, 37]. The PRISM (Precision Implantable Stimulator for Migraine) study conducted by Lipton et al., only available in abstract form, failed to show a significant difference in the reduction of migraine days/month between the active and sham groups after 12 weeks (−5.5 vs. 3.9 days/month, *p* = 0.29) [17]. Saper et al. published results of the ONSTIM (Occipital nerve stimulation for the treatment of intractable chronic migraine headache) trial in 2010 [30]. Of the 77 patients included, all subjects had failed to respond to at least two different classes of medication and had reported positive response to greater occipital nerve block. Various outcome measures including reduction in headache days, pain intensity and pain duration were numerically superior in the treatment group. A responder was defined in this study as a subject reporting a more than 50 % reduction in monthly headache days or a more than three-point reduction in average pain intensity. Three-month responder rates were 39 % for the active group, 6 % for the sham-control group and 0 % for the medical management group. The percentage reduction in severe headache days a month was 24.4 % (±43.6) for the active group and 10.3 % (±34.0) for the sham group, corresponding to a reduction of 5.1 days (±8.7) a month in the active group and 2.2 (±6.4) in the sham group. The largest randomized sham-controlled study on ONS in CM was conducted on 157 subjects by Silberstein et al. [34]. This group failed to find a significant difference in the primary outcome measure (those achieving a more than 50 % reduction in average pain intensity at 12 weeks) between active (17.1 %) and sham (13.1 %) stimulation groups. However, a number of secondary outcomes did suggest that ONS had a benefit including the numbers achieving a 30 % reduction in pain severity and a 30 % reduction in headache frequency. Long-term open-label follow-up of this cohort for a total of 12 months revealed that there was a significant reduction in the number of headache days (defined as days with more than 4 h of moderate-to-severe pain) of 6.7 days (±8.4) [10]. The percentage reporting a more than 50 % reduction in headache days and/or pain intensity was 47.8 %. Pooled results from these three trials show that ONS is associated with a mean reduction of 2.59 moderate-to-severe headache days a month (95 % CI 0.91, 4.27) after 3 months treatment compared with sham control [8].

Several open-label series have been published suggesting efficacy of ONS in CM, however, many of these have been of small numbers with restricted follow-up. Two centers analyzed their long-term data in retrospective reviews. Brewer et al. reported 12 CM patients with average follow-up of 34 months (range 1–70 months) and Palmisani et al. reported 19 CM patients with average follow-up of 10 years (range 1–19 years) [5, 29]. Outcome in both was based on the patients subjective reporting via a telephone interview. In the Brewer series, five patients (41.6 %) reported a more than 50 % overall benefit whereas nine (47.3 %) of the Palmisani series reported a more than 50 % reduction in pain intensity and/or frequency.

Our uncontrolled, open-label, prospective observational study showed that in highly refractory CM patients with prolonged follow-up, ONS resulted in a significant reduction of moderate-to-severe headache days a month (8.51 days). In total, 45.3 % of patients showed a more than 30 % reduction of monthly moderate-to-severe headache days following treatment. Five patients were completely pain free and had been for prolonged periods. Significant improvements were also seen in pain intensity, daily pain duration and headache related disability. Although our response rate is below that of the open label series quoted above, our outcome measure is objective and from prospectively completed headache diaries and is thus a more robust measure. The use of a 30 % improvement in outcome measure is accepted in the chronic pain literature as representing a “much improved” clinical state and has been accepted by both the International Headache Society clinical trials subcommittee as being a realistic and clinically relevant improvement in those with chronic migraine [10, 11, 33]. With this is mind and with such a complex group of patients present in this cohort, we feel that a 30 % improvement level is justified.

The primary outcome measure of moderate-to-severe headache days was chosen in accordance with the 2008 guidelines for controlled trials of prophylactic treatment of chronic migraine in adults produced by the Clinical Trials Subcommittee of the International Headache Society [33]. However, a wide variety of headache outcome measures (e.g. “headache days”, pain intensity) have been used in previous case series of ONS for CM and so direct comparison between studies can be somewhat difficult. With this in mind, there is an obvious need for a consensus on the most appropriate outcome measures for ONS efficacy. Our group is also more complicated than those in the previous series with many suffering multiple chronic headache conditions, reporting a higher number of failed past medications and with 94.3 % of them recording background or interictal pain. In such an intractable group of patients, a continued response in over one-third of patients after such prolonged follow-up should be viewed with cautious optimism.

The rates of serious adverse event in our series was below that of previous reported groups. In the randomized trials, concerns were raised over the high rates of lead migration and infection. The ONSTIM trial quoted rates of lead migration at 24 % and infection in at least 18 % of patients, whilst the Silberstein study reported rates of 19 and 7 %, respectively. Our group had no episodes of lead migration and only a single episode of mild wound site infection treated with oral antibiotics. Our implants were conducted by a single highly skilled surgical team and our results mirror those found by Sharan et al. in describing high levels of implanter experience being associated with significantly lower levels of complications [32]. Recent guidelines recommending that ONS should only be carried out in a limited number of highly specialized centers should lead to improvements in adverse event rates and a reduction in the current discrepancy between centres.

Our group do not conduct trial-periods of stimulation as is carried out in a number of other centres. The intention of such trials is that they will positively select those most likely to respond to long-term ONS treatment and they have in-fact been used as inclusion criteria for a number of controlled trials. However, it is clear that a positive trial does not guarantee longer-term success. In the study by Silberstein et al., despite all subjects having a positive trial period, the study still failed to reach its primary endpoint [34]. Other open-label series support the view that trial stimulation does not predict success. Palmisani et al. had a trial success rate of 88 % but removed 7/23 systems implanted due to lack of efficacy and Brewer et al. reported high trial success rates (89 %) but a long-term benefit in only 42 % CM patients [5, 29]. A study using longer trial-periods of one month has also failed to show an association between trial response and ONS outcome [28]. Our data suggests that there is a delay in patients reporting clinical effect of ONS which may be up to 6 months in responders. This may explain why trial stimulation does not predict success and suggests that the early response reported by some groups may be due to a placebo effect. The hypothesis that the neuromodulatory effects of ONS are due to slow, plastic changes within the pain-structures of the brain is supported by our data on time to clinical effect and observation of gradual return of pain when ONS is stopped. This hypothesis of plastic-change is in direct conflict to the rapid action seen in trial stimulation. Given that response rates in those groups using trial stimulation are similar to our patients who do not undergo such trials, we do not feel that the current evidence supports the use of trial stimulation and the additional surgical risks this entails. This remains a controversial area and we support further investigation into the true predictive value of trial stimulation in ONS.

This is the first study on ONS in CM to include a significant proportion of patients with multiple headache phenotypes. Nearly a third of our cohort had other headaches in addition to CM, all carefully phenotyped by headache specialists and all recorded in separate headache diaries to allow outcomes to be differentiated. It is often speculated that those with multiple chronic headaches may have a worse response to treatments than those with a single phenotype, however, there is no published data supporting this. Occipital nerve stimulation has been employed to treat a number of primary headache conditions and it may be that the above view does not apply to this particular treatment modality as a single implant can potentially improve multiple conditions. Although numbers were small, we did compare outcomes between those with and without multiple headache types at each time point. Interestingly, the only time point at which there was a significant difference between the responses was at the point of final follow-up where those with multiple phenotypes appeared to have a significantly better response than those with CM alone (30 % response rate of 66.7 vs 34.3 %; *p* = 0.012). The reason for this discrepancy is unclear but likely due to a combination of numbers in the two groups being too small to either show a real difference at individual time points or a confounding factor of a wide range of follow-up times being included in the primary outcome measure of final follow-up point. On further examination, the distribution of final follow-up times is not normalized and this may influence the result at this point. This point obviously needs clarification with data being collected from larger cohorts but also with a well-controlled study comparing those with multiple phenotypes to those with the same phenotypes in isolation. Our current data does not support the concept that those with multiple headache types respond poorly to ONS and thus such patients should not be deprived of the treatment. In fact, ONS is a good example of one procedure able to treat multiple conditions.

Assessments of headache related disability and quality of life showed numerical improvements but only HIT-6 and EQ-VAS showed any statistical improvement in the cohort as a whole. In a subgroup analysis, however, those with a positive response were found to have statistically significant reductions in a variety of quality of life and affect measures which were not mirrored in the non-responder group. A failure to observe significant change across all assessments despite improvements in headache frequency is reported in cases of epilepsy surgery and spinal cord stimulation and has been attributed to a “burden of normality” [1, 20]. Given that 33.9 % also suffered other headache types that did not necessarily respond completely to ONS and that nearly all patients continued to have migraine pain of some level, they will still exhibit a disability burden from their pain, even if their migraine has significantly improved. This theory is supported by a lower reduction in disability scores in the multiple phenotype group compared to the CM alone group of patients. Recently, Clark et al. reported on the long term functional outcomes of combined supra-orbital and occipital nerve stimulators for CM [9]. The group found that improvements in functional outcome (MIDAS and BDI) were only significant during the first 6-months post implant but not after prolonged follow-up (average 44.5 months). They speculated that this was due to the loss of a “honeymoon period” and as yet unexplained complex interactions between pain and functional status. Our cohort seems to suggest that this is not necessarily accurate as even after a follow-up period of nearly 4 years, over a third of patients still reported clinical response and significantly reduced headache disability.

The strengths of this study include the large sample size, the long follow up period and, importantly, the prospective nature of the data collection (a first in long-term observational ONS cohorts of CM). The real-life nature of the data is also valuable. Patients were not subjected to the strict inclusion criteria of a study and represent the types of highly complex CM patient typically seen in specialized neuromodulation centers. The limitations of this study are mainly centered on the lack of a placebo or sham-stimulation group. However, it is most unlikely that our observations can be explained by placebo alone. We found that there was a delay of months to reporting clinical effect (5.50 months) and a delay before pain worsened when the device was off (2.50 months). These observations are reproducible across multiple ONS cohorts for a variety of primary headache disorders and argue against a pure placebo effect [6, 16, 19]. This time delay may also explain why shorter trials, reporting at 3-months post-implant, do not mirror the more favorable open-label clinical experience of ONS in CM. Other factors against a pure placebo response include the previous intractable nature of the group, a stable response with long-term follow up and the previously quoted placebo rates of between 6 and 13 % in the controlled trials of ONS in CM being below the 45.3 % response rate we quote here [30, 34]. The extrapolation of 2-weeks diary data to represent a month is not ideal but the time-span was chosen as it is the normal diary kept by all of our patients seen in clinic, with or without ONS. The data was collected from patients in a real clinical environment and we are aware that asking for too much information may lead to patients being unable to comply with requirements. Therefore, a 2-week diary was chosen to ensure high rates of compliance and diary completion. This method may be more at risk of being influenced by natural fluctuations in CM severity, however, in our cohort of highly refractory patients such fluctuations were not commonly seen prior to ONS. In the future, consensus on the most relevant outcome-measures for ONS and the development of electronic-diaries may improve ease of dairy keeping and allow long periods of data to be collected easily.

The high levels of complications requiring surgical intervention published in the literature have led to concerns over the cost-effectiveness as well as safety of the procedure. The current equipment used for ONS is designed for spinal cord stimulation and not intended for implantation in the occipital region. Advances in technology have already led to reductions in intervention rates, for example a reduction in need for battery replacement with rechargeable IPG development, and hopefully ONS specific equipment may be available in the future. However, it must be noted that the one company (St Jude Medical) who were granted a European CE Mark Approval for the use of their ONS to treat CM in 2012 had that approval removed in 2014 as it was felt that there was not enough data to demonstrate that the benefits outweighed the risk of therapy [35]. These concerns are a major issue in neuromodulation for migraine and raise the need for high quality, well planned, large placebo-controlled trials to look at efficacy and safety in the long-term treatment of CM.

## Conclusion

In this uncontrolled, open-label prospective observational study with long-term follow-up of efficacy, functional outcome and safety of ONS in highly intractable complex CM patients, over 40 % of patients reported sustained clinical benefit after a mean follow-up of 4 years. Sustained benefit was seen even in those with multiple headache types in addition to CM. Responders showed improvements in functional outcomes and headache related disability. Adverse event rates are low when implants are conducted in specialist centers. There appears to be a time delay of up to 6 months before clinical effect of ONS is seen which calls into question the practice of trial stimulation prior to implant. There are still concerns over the risk to benefit ratio and cost effectiveness of ONS despite positive open-label data and a well-designed double-blind controlled trial with long-term follow-up is needed to clarify the position of neuromodulation in chronic migraine.

## Abbreviations

BDI-II, Becks Depression Inventory; CCH, chronic cluster headache; CM, chronic migraine; CI, confidence interval; EQ5D, Euro-QoL 5D index; EQ-VAS, Euro-QoL visual analogue score; HIT-6, headache impact test 6; HAD-A, hospital anxiety score; HAD-D, hospital depression score; IPG, implantable pulse generator; ICHD, International Classification of Headache Disorders; MIDAS, migraine disability assessment score; ONS, occipital nerve stimulation; ONSTIM, occipital nerve stimulation for the treatment of intractable chronic migraine headache; PRISM, precision implantable stimulator for migraine; VRS, verbal rating scale

## Additional file

Additional file 1: Figure S1. Examples of headache diaries used throughout the study. Example of headache diaries in a patient with both chronic migraine and chronic cluster headache. The patient has been asked to record her migraine pain severity on VRS scale 0–10 at every hour during the day. Note that on first diary, patient has recorded 2 cluster attacks at around 0750 and 1900. These are replicated on her separate cluster attack diary shown in Figure S1b. The use of separate headache diaries for each phenotype allowed patient and investigators to ascertain outcome for each phenotype. a: Example of chronic migraine diary. b: Example of cluster attack diary. (DOCX 128 kb)
