# Supplementary material for: Long-term outcomes of occipital nerve stimulation for chronic migraine: a cohort of 53 patients
Source: J Headache Pain. 2016 Jul 30;17(1):68. doi: 10.1186/s10194-016-0659-0 (PMC4967414; doi:10.1186/s10194-016-0659-0)
Supplement: Additional file 1: Figure S1. — Examples of headache diaries used throughout the study. Example of headache diaries in a patient with both chronic migraine and chronic cluster headache. The patient has been asked to record her migraine pain severity on VRS scale 0–10 at every hour during the day. Note that on first diary, patient has recorded 2 cluster attacks at around 0750 and 1900. These are replicated on her separate cluster attack diary shown in Figure S1b. The use of separate headache diaries for each phenotype allowed patient and investigators to ascertain outcome for each phenotype. a: Example of chronic migraine diary. b: Example of cluster attack diary. (DOCX 128 kb) [file 10194_2016_659_MOESM1_ESM.docx]

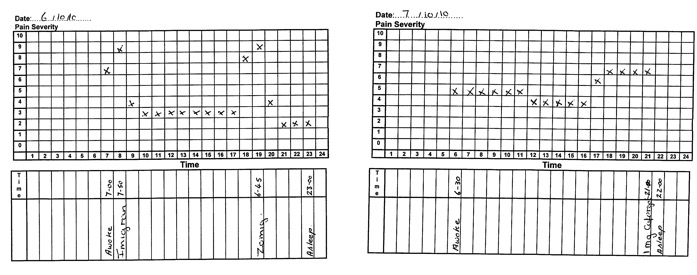


**Figure S1a**


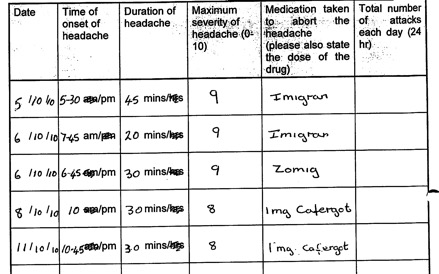


**Figure S1b**

**Figure S1: Examples of headache diaries used throughout the study**

**Legend Figure S1:** Example of headache diaries in a patient with both chronic migraine and chronic cluster headache. The patient has been asked to record her migraine pain severity on VRS scale 0-10 at every hour during the day. Note that on first diary, patient has recorded 2 cluster attacks at around 0750 and 1900. These are replicated on her separate cluster attack diary shown in Figure S1b. The use of separate headache diaries for each phenotype allowed patient and investigators to ascertain outcome for each phenotype.

**Figure S1a:** Example of chronic migraine diary

**Figure S1b**: Example of cluster attack diary
